# The Power of Tolerance vs. Unselfishness as a Cultural Determinant of Cooperation

**DOI:** 10.3389/fpsyg.2021.678237

**Published:** 2021-09-07

**Authors:** Kimmo Eriksson, Brent Simpson, Irina Vartanova

**Affiliations:** ^1^School of Education, Culture and Communication, Mälardalen University, Västerås, Sweden; ^2^Institute for Futures Studies, Stockholm, Sweden; ^3^Department of Sociology, University of South Carolina, Columbia, SC, United States

**Keywords:** tolerance, unselfishness, cooperation, norms, metanorms, cultural variation

## Abstract

Cooperation in collective action problems and resource dilemmas is often assumed to depend on the values of the individuals involved, such as their degree of unselfishness and tolerance. Societal differences in cooperation and cooperative norms may therefore result from cultural variation in emphasis on these personal values. Here we draw on several cross-national datasets to examine whether society-level emphasis on unselfishness and tolerance and respect for other people predict how societies vary in cooperation [in a continuous prisoner’s dilemma (PD)] and in norms governing cooperation [in a common pool resource dilemma (CPR)]. The results suggest that high levels of cooperation and cooperative norms are promoted specifically by a cultural emphasis on tolerance.

## Introduction

What makes some societies more cooperative than others? This question is important for a number of reasons, including the possibility that societal differences in cooperation may have played an important role in human cultural evolution (Henrich, 2004; Richerson et al., 2016). Various approaches to the problem view international differences in cooperation as governed by social norms about cooperating and sanctioning non-cooperators (Chudek and Henrich, 2011), or as being driven by more domain general factors that are less specifically tied to the cooperative domain, such as generalized trust (Yamagishi and Yamagishi, 1994; Balliet and Van Lange, 2013) and relational mobility (Romano et al., 2021). Here we focus on another domain-general factor: the kind of personal qualities that a society emphasizes and values in its citizens. Specifically, we focus on the qualities of unselfishness and tolerance. While prior work leads straightforwardly to the hypothesis that higher societal valuations of unselfishness will lead to more cooperation, we explain why tolerance can be expected to be at least as important. We then report findings showing that a societal emphasis on tolerance plays a stronger role than an emphasis on unselfishness in predicting international differences in cooperation, cooperative norms, and cooperative metanorms.

### Cooperation: Behavior, Norms, and Metanorms

This paper focuses on how societal emphasis on unselfishness and/or tolerance may support a suite of behaviors underlying cooperation, including cooperative behavior, cooperative norms, and cooperative metanorms. We study these behaviors in the context of cooperation problems, which are situations that pose a conflict or tension between individual and collective interests (Van Lange et al., 2014; Simpson and Willer, 2015). These situations involve two or more persons, each of whom can choose to act in a way that benefits others at some cost to themselves (i.e., “to cooperate”). The most often studied cooperation problem is the prisoner’s dilemma (PD), in which two agents make simultaneous decisions about whether to make a personal sacrifice to give the other a (larger) benefit. Public goods dilemmas (PGDs) have a similar incentive structure but entail more than two agents. Another commonly studied social dilemma is the common pool resource dilemma (CPR), where agents must harvest from a commonly owned resource pool judiciously in order to not leave other agents bereft. We refer to the behavioral measures obtained from studies of PDs and CPRs as cooperative behaviors. Previous research, including a study of cooperative behavior in a PGD in 14 societies (Herrmann et al., 2008) and a recent PD experiment in 42 societies (Romano et al., 2021), shows clear evidence of cross-national differences in cooperative behavior.

Social norms have long been known to be an important determinant of behavior in cooperation problems (Bicchieri, 1990; Fehr and Fischbacher, 2004; Irwin and Simpson, 2013). But researchers have also argued that norms governing cooperation are not sufficient without “metanorms” for how norm violations should be dealt with (Axelrod, 1986, 1997; Horne, 2001). Although much of this work has focused on metanorms prescribing that peers should punish non-cooperators, such responses may also hurt cooperation by inviting retaliation and deepening conflicts within the group (Nikiforakis, 2008; Nikiforakis and Engelmann, 2011). A more crucial aspect of metanorms may therefore be to prescribe which form the response to a norm violation should take (Eriksson et al., 2021).

Norms and metanorms can be measured in various ways (Horne and Mollborn, 2020). An approach used in the aforementioned cross-national study of public goods game by Herrmann et al. (2008) is to examine the extent to which participants use costly punishment in response to others’ decisions to cooperate or not. To disentangle norms and metanorms, another paradigm uses an animation of a non-cooperator who harvests the entire common pool resource, leaving the other agents with nothing. Participants rate the appropriateness of the non-cooperator’s behavior as well as the appropriateness of various responses to it (Eriksson et al., 2016). The simple animations are designed to be easily comprehensible to laypeople and are especially suitable for cross-cultural research (Eriksson et al., 2017).

The animation paradigm was used in the International Study of Metanorms (ISMN), a recent study of metanorms across 57 societies (Eriksson et al., 2021). The ISMN found that metanorms prescribe different responses in different societies. Specifically, societies with higher median income were more approving of gossip about norm violators, but less approving of physical confrontation against norm violators. In line with these findings, prior work shows that gossip is a non-costly mechanism for strengthening cooperative norms and promoting cooperative behavior (Feinberg et al., 2012; Wu et al., 2015; Imada et al., 2020). Physical confrontation, by contrast, is an aggressive response that may worsen conflicts within a group, and people subjected to aggression may become less inclined to cooperate (Rothmund et al., 2011). Thus, cooperation should be promoted by metanorms against the use of physical confrontation and in favor of the use of gossip.

In sum, we conceive of a society’s cooperativeness as a phenomenon comprising three levels: (i) actual cooperative behavior, (ii) norms supporting cooperative behavior, and (iii) metanorms that, by favoring gossip about norm violators while disfavoring physical punishment, support cooperative norms while avoiding escalating conflicts. The first aim of this paper is to validate this multi-level conception of cooperativeness. The second aim is to examine whether cooperative societies are characterized by a cultural emphasis on certain personal qualities.

### Unselfishness vs. Tolerance

Prior work, using data from the World Values Survey (WVS) and European Values Survey (EVS), has established cultural variation in the emphasis placed on children developing specific personal qualities (Inglehart, 2006). While prior work has investigated a wide range of personal qualities, here we focus on how societies value unselfishness and tolerance and respect for other people, which we often abbreviate to “tolerance.” As detailed below, there are reasons to expect both these personal qualities to be highly relevant to cooperation. Moreover, previous work has shown that valuations of these qualities vary quite independently across societies (e.g., Mendez, 2015; Berggren et al., 2019), and may therefore have mutually independent effects on cooperation.

Much of the cooperation literature takes for granted that non-cooperation amounts to a selfish choice. From this perspective, unselfishness should be a key determinant of voluntary cooperation. Indeed, the large literature on social value orientation shows a strong relation between unselfishness and cooperation (Van Lange, 1999; Bogaert et al., 2008; Balliet et al., 2009). For this reason, one might expect societies that put a high value on unselfishness to have a high level of cooperativeness. However, several features of cooperation problems make it unclear whether fostering unselfishness is in fact a feasible pathway to higher cooperativeness in society at large. Voluntary cooperation is built on two key foundations, trust and trustworthiness. More specifically, voluntary cooperation generally requires not only an interest in the welfare of others and a reluctance to exploit others’ cooperation (trustworthiness). It also depends on a belief that others will not exploit one’s cooperation (trust). While unselfishness should be a strong predictor of trustworthiness, a belief that one should take others’ interests and outcomes into account (unselfishness) is conceptually distinct from a belief that they will take one’s own into account (trust). Therefore, fostering unselfishness may not be sufficient to raise the overall cooperativeness level in society.

The link between unselfishness and cooperative behavior is further weakened by research showing that unselfishness is generally parochial, rather than universal. As an example, de Dreu (2010) found that, compared to their proself counterparts, those with a prosocial value orientation trusted and cooperated with ingroup members more, but were not more trusting and cooperative toward outgroup members. Finally, where there is heterogeneity in the level of unselfishness or interest in a given cooperative outcome (e.g., the production of a public good or preservation of a commonly held resource), those who are more selfish or who are less interested in a given collective goal can drive down overall cooperation levels over time. For instance, research shows that this heterogeneity can lead to an unraveling of cooperation, as initially unselfish agents start by cooperating at high rates but then withdraw their contributions in response to low contributions by others (Sell and Wilson, 1991; Ostrom, 2000). This unraveling is even more likely in “noisy” situations (Van Lange et al., 2002; Klapwijk and Van Lange, 2009) where, due to personal or cultural differences or other features of the environment, intentions to cooperate may be misread as non-cooperation. Thus, both societal heterogeneity (in a variety of forms) and the fact that voluntary cooperation is founded on both trustworthiness and trust may limit the role of societal valuations of unselfishness in voluntary cooperation.

We suggest that a cultural focus on tolerance and respect for other people might overcome these limitations. That tolerance may drive cooperation has received extensive attention in the animal literature (e.g., Melis et al., 2006; Massen et al., 2015), but much less in the social sciences. What little work does exist, however, is suggestive. Economists have linked variation in tolerance to economic development (Florida, 2005) and human capital and wages (Florida et al., 2008). Further, tolerance is strongly associated with economic globalization (e.g., trade and investments) and social globalization (e.g., personal contacts and information flows), which are related to cooperation (Berggren and Nilsson, 2015). Perhaps most importantly, international differences in tolerance are associated with support for the welfare state, a societal-level public good (Crepaz and Damron, 2009).

Why might a cultural emphasis on tolerance predict societal level cooperation and norms buttressing cooperation? Here we note that tolerance may be less subject to some of the potential weaknesses identified in our discussion of unselfishness and cooperation. First, tolerance may help widen the “radius of trust” (Fukuyama, 1995; Delhey et al., 2011; van Hoorn, 2014), i.e., whether or not one’s trust is limited to family and close friends or whether it extends to strangers and outgroup members. An emphasis on greater tolerance and respect for other people, including those who have different views and beliefs or who are from different backgrounds, should be associated with a wider radius of trust, i.e., a baseline trust in strangers and outgroup members. Similarly, tolerance may lead to a greater willingness to forgive relatively minor transgressions in noisy environments (or where cross-cultural communication leads to misunderstanding) and prior research demonstrates that such forgiveness can prevent the unraveling of cooperation (Van Lange et al., 2002; Klapwijk and Van Lange, 2009). These arguments show that cooperation based on tolerance may be more sustainable than cooperation based on unselfishness. In further support of this line of argument, a simulation study of a spatial public goods game found that the presence of tolerant strategies tends to promote cooperation (Szolnoki and Perc, 2016).

In addition to driving cooperative behavior, note that societal valuations of tolerance (and unselfishness) may also shape norms and metanorms supporting cooperation. Indeed, communicating values of tolerance and unselfishness can be seen as the transmission of prosocial norms. A study that emphasized the distinction between prosociality and unselfishness explicitly operationalized prosociality as “tolerance and respect for other people” (Van de Vliert et al., 2009). It is further plausible that tolerance and respect for other people includes the kind of metanorms that avoid escalation of conflicts.

### Other Predictors of Societal Differences in Cooperation

Previous research points to several other potential predictors of societal differences in cooperation, a number of which were included in recent studies of international differences in cooperation (Romano et al., 2021) and metanorms (Eriksson et al., 2021). The strongest predictors of cooperation in Romano et al.’s study were measures of low historical prevalence of infectious diseases and high societal relational mobility. A slightly lower correlation was obtained between cooperation and the cultural dimension known as indulgence (i.e., perceptions of happiness, life control, and importance of leisure), which has previously been shown to predict societal variation in giving (Guo et al., 2018). Interestingly, Romano et al. did not find that cooperation levels correlated with a society-level measure of trust, despite cooperation being known to relate to trust at the individual level (Balliet and Van Lange, 2013). Strong predictors of cooperative metanorms in the ISMN included median income and the cultural dimension known as individualism (Eriksson et al., 2021). We therefore assess how our key explanatory variables (societal emphasis on unselfishness and tolerance) fare in predicting society-level measures of cooperation relative to historical prevalence of infectious diseases, societal-level relational mobility, indulgence, trust, median income, and individualism.

### Outline of Study

Our first aim is to examine whether cooperative behavior, cooperative norms, and cooperative metanorms vary across societies in a consistent way, such that more cooperative societies also have more cooperative norms and cooperative metanorms. To do so, we collate data from the aforementioned multi-society studies of Romano et al. (2021) and Eriksson et al. (2021). Given these society-level measures of cooperation, our second aim is to examine whether they are predicted by cultural valuations of unselfishness and tolerance, and how these predictors compare to the other predictors discussed in the preceding section. A third aim is to tease out individual-level effects of valuations from the genuinely society-level effects. Our arguments about the possible impact of unselfishness and tolerance on cooperation are centered on society level phenomena: the accumulated effect of people impressing behaviors and norms on other people. Note that an individual’s values are also likely to drive their own behaviors’ that is, we should expect that those who value unselfishness or tolerance in others are themselves likely to be relatively unselfish or tolerant, respectively. In our empirical analysis we will therefore attempt to tease out the genuine society-level effect from the individual-level effect. To achieve this, we analyze individual-level data on norms, metanorms, and valuations of unselfishness and tolerance from the ISMN (Eriksson et al., 2021).

## Materials and Methods

For our main analyses, we examine data for 62 societies for which we had valuations of unselfishness and tolerance from the WVS/EVS and at least one measure of cooperation (cooperative behavior, norms, or metanorms). For these 62 societies we also collated data on historical prevalence of infectious diseases, societal relational mobility, median income, indulgence, individualism, and trust. The final dataset and analysis code are publicly available at https://github.com/irinavrt/tolerance-vs-unselfishness. For the subsequent individual-level analysis, we instead examine data from the ISMN, which include valuations of unselfishness and tolerance as well as measures of cooperative norms and metanorms for 17,888 participants in 57 societies.

### Societal Emphasis on Unselfishness and Tolerance

The WVS is a publicly available survey of values administered to nationally representative samples.^1^ It has been conducted in seven waves since the 1980s with a new wave roughly every 5 years. Many items are shared with the European Values Study (EVS), which is similar in format to the WVS and also publicly available.^2^ Thus, researchers often merge data from these two surveys (Berggren and Nilsson, 2015). We use the most recent year of WVS or EVS in which the society participated^3^ (sample sizes ranged from 999 to 3,531 with a mean of 1,592).

In the WVS/EVS, valuations of personal qualities are measured by a single question: “Here is a list of qualities that children can be encouraged to learn at home. Which, if any, do you consider to be especially important? Please choose up to five!” Respondents could select up to five from a list of qualities including “unselfishness,” “tolerance and respect for other people,” and several additional options that are presumably less related to cooperation (e.g., “obedience” and “determination”). Data based on this question have been used as a measure of values in a large number of papers across the social sciences, including economics (Lindbeck and Nyberg, 2006), sociology (Inglehart and Baker, 2000), education (Mendez, 2015), and cultural psychology (Minkov and Hofstede, 2012). We calculate the percentages of respondents in a society that selected “unselfishness” and “tolerance and respect for other people,” applying the sampling weights provided with the WVS and EVS datasets. These percentages constitute our measures of how much unselfishness and tolerance are valued in different societies. This measure was also included in the ISMN (Eriksson et al., 2021). We analyze those data separately, as detailed below.

### Cooperative Behavior

The PD and similar game theoretic situations are commonly used to measure cooperation (Balliet et al., 2011). A continuous version of the PD was used in a recent online experiment with more than 18,000 participants in 42 societies (Romano et al., 2021). Participants in this experiment were assigned partners with which they played the PD. Across treatments, partners were either from the same nation, a set of outgroup nations, or an unidentified stranger. Each participant played the game 12 times, each time with a different partner, yielding 12 decisions per participant (we use the average of these decisions). In each game, both participants were given 10 Monetary Units (MUs) and made simultaneous decisions on how many MUs to keep for themselves and how many to give to their partner. Participants were informed that the amount given to their partner was doubled and that their partner simultaneously had the option to give any amount (of 10 MUs) to them, which would also be doubled (see Romano et al. for further details). Participants thus faced a PD (or, more generally, a social dilemma) because the individually rational strategy is to keep the entire endowment, but both participants earn more if they overcome this temptation and “cooperate” with one another.

We use data from Romano et al. (2021) to measure the level of cooperation in a society. As the design with different treatments was identical in each society, we use the average percentage of the endowment that was given to partners across all treatments and all participants from a society.^4^ This measure was available for 40 of the 62 societies in our study.

### Cooperative Norm and Metanorms

The ISMN (Eriksson et al., 2021) is a study of norms and metanorms conducted with more than 22,000 participants in 57 societies. Norms and metanorms were measured in relation to a number of different norm violation scenarios. Here we focus on the cooperation scenario, an abstract animation of a CPR used in previous research of norms surrounding cooperation and peer punishment (Eriksson et al., 2016, 2017). The agents in this animation are four triangles of different colors (Blue, Green, Pink, or Purple) based in different corners of a white space. In the center of the space is a collection of small circles, depicting a common resource. The animation starts with the triangles taking turns at harvesting circles, one at a time, by moving the circle to their own corner. After a while, Purple violates the norm by harvesting all the remaining circles in one go. Thus, the animation shows Purple as a non-cooperator. Participants judged the appropriateness of Purple’s action on a six-point scale from extremely inappropriate (here coded 5) to extremely appropriate (coded 0). To control for response sets, appropriate ratings were standardized for each respondent (i.e., calibrated so that the mean rating across 50 appropriateness items in the survey is the same for all respondents, see Eriksson et al., 2021). Following Eriksson et al. (2021), we measure the strength of the cooperation norm (i.e., the norm against non-cooperation) in a society as the society-mean rating of the non-cooperator.

To measure the metanorm against physical confrontation, a second part of the animation showed Blue discovering the norm violation and reacting by going to Purple’s corner for a physical confrontation that makes Purple lurch backward. Participants judged the appropriateness or inappropriateness of Blue’s action on the same scale as before. We use the society-mean standardized rating as a measure of the strength of the metanorm against physical confrontation.

Participants were also asked to consider the appropriateness of using gossip (i.e., speaking to others) as a response to Purple’s action. Ratings were given on the same scale and standardized, as in the above ratings. After reverse-coding, so that high values represent appropriateness, we use the society-mean rating as a measure of the strength of the metanorm in favor of using gossip.

These measures of cooperative norms and metanorms were available for 51 of the 62 societies in our study.

### Other Predictors of Cooperation

We obtained measures of the historical prevalence of infectious diseases for all 62 societies from Murray and Schaller (2010), here reverse-coded so that high values signify absence of pathogens. We obtained measures of societal relational mobility for 31 societies from Thomson et al. (2018), measures of median per-capita income for 60 societies from a Gallup study (Phelps and Crabtree, 2013), and measures of indulgence (59 societies) and individualism (62 societies) from the Hofstede Insights website.^5^ Finally, our measures of self-reported trust from all 62 societies come from the WVS and EVS data.

### Individual-Level Data

In our second set of analyses, we turn to the ISMN (Eriksson et al., 2021) as our data source on valuations of unselfishness and tolerance. The ISMN includes data on valuations of unselfishness and tolerance and measures of cooperative norms and metanorms for 17,888^6^ participants in 57 societies. As described above, the individual-level data on valuations are binary while the individual-level data on norms and metanorms are integers between 0 and 5.

### Analysis Plan

This study of secondary data was not preregistered. We keep our analyses as simple and straightforward as possible. In the analyses of society-level data, we focus on raw correlations between societies’ valuations of unselfishness and tolerance and their cooperativeness. We compare these correlations with those obtained with other predictors of cooperativeness. In the multi-level analysis, we include only valuations of unselfishness and tolerance (at the society and individual levels) as predictors of measures of cooperativeness.

## Results

We divide the results section in two parts. The first part reports society-level analyses using data for 62 societies from multiple sources. The second part reports multilevel analyses using individual-level data for 57 societies from one single source.

### Society-Level Analysis Using Data From Multiple Sources

Descriptive statistics of all society-level variables are reported in Table 1. Several aspects of these descriptives are noteworthy. For instance, tolerance was overall more highly valued than unselfishness; on average the difference was 37.3 percentage points. Cooperation in the PD ranged from an average of a third to a half of endowments being given to partners. The cooperative norm in the common pool resource scenario was generally strong (non-cooperation receiving a mean inappropriateness rating of close to the high end on the scale from 0 to 5). Despite the high level of disapproval of non-cooperation, physical confrontation tended to be rated as an inappropriate response to the non-cooperator (receiving a mean rating clearly above the scale midpoint, on the inappropriate side). Gossip was generally rated as neither inappropriate nor appropriate (receiving a mean appropriateness almost exactly on the scale midpoint).

**TABLE 1:** Descriptive statistics of key variables.

**Variable**	**N**	**Min**	**Max**	**M**	**SD**
Valuation of unselfishness	62	4.0	54.9	29.0	12.3
Valuation of tolerance	62	31.7	93.7	66.3	13.4
Cooperative behavior in PD	40	33.8	49.5	41.6	3.7
Cooperative norm in CPR	51	3.7	4.4	4.1	0.2
Metanorm against physical confrontation	51	2.2	4.0	3.2	0.4
Metanorm in favor of gossip	51	1.8	2.8	2.4	0.2
Absence of infectious diseases	62	−1.2	1.2	0.0	0.6
Societal relational mobility	31	−0.4	0.4	0.0	0.2
Median income (thousands of dollars)	60	0.0	18.6	5.5	4.9
Indulgence	59	0	100	46.7	23.0
Individualism	62	8.0	91.0	41.1	23.8
Trust	62	0.0	0.7	0.3	0.2

We hypothesized that cooperative behavior, a cooperative norm, and metanorms in favor of gossip and against physical confrontation would cohere. Consistent with this hypothesis, all pairwise correlations were positive (ranging between 0.34 and 0.56) and confidence intervals do not include zero, see Table 2. However, confidence intervals are provided as a guide only and should not be taken at face value. The reason is that the units of analysis (societies) do not satisfy the key statistical assumptions; they are neither a random sample of all possible countries, nor fully independent. For the same reason we do not report *p*-values.

**TABLE 2:** Pearson correlations between cooperative behavior, cooperative norms, and cooperative metanorms.

	**(1)**	**(2)**	**(3)**
Cooperative behavior			
Cooperative norm	0.34 [0.03, 0.58]		
Metanorm against physical confrontation	0.56 [0.33, 0.75]	0.54 [0.28, 0.72]	
Metanorm in favor of gossip	0.40 [0.04, 0.63]	0.38 [0.02, 0.65]	0.45 [0.14, 0.69]

*Pearson correlations based on *n* = 29 societies in column (1) and *n* = 51 societies in the columns (2) and (3). 95% BCa confidence intervals, based on 2,000 bootstrap samples, within brackets.*

To obtain a cooperation measure for all 62 societies we first *z*-score transformed all four measures to a common scale. We then calculated a cooperation index by taking the mean of the scores available for a given society, which meant four scores for 29 societies, α = 0.73; three scores (norm and metanorms) for 22 societies, α = 0.72; and only the cooperative behavior score for 11 societies.

Consistent with previous literature, valuations of unselfishness and tolerance were uncorrelated, *r* = 0.07, 95% CI [−0.19, 0.31], *n* = 62. We next calculated pairwise Pearson correlations between these valuations and all other predictors of cooperation (see Table 3). A clear pattern emerged: Valuations of unselfishness correlated only weakly, if at all, with other predictors, whereas valuations of tolerance correlated at least moderately strongly with all predictors.

**TABLE 3:** Pearson correlations between valuations of personal qualities and other predictors of cooperation.

**Other predictor**	**N**	**Correlation with valuation of unselfishness**	**Correlation with valuation of tolerance**
Absence of pathogens	62	−0.12 [−0.36, 0.15]	0.29 [0.01, 0.50]
Relational mobility	31	0.29 [−0.02, 0.51]	0.37 [0.10, 0.57]
Median income	60	−0.01 [−0.28, 0.25]	0.55 [0.32, 0.71]
Indulgence	59	0.24 [−0.00, 0.47]	0.44 [0.21, 0.60]
Individualism	62	0.17 [−0.09, 0.41]	0.41 [0.15, 0.60]
Trust	62	0.10 [−0.13, 0.30]	0.43 [0.18, 0.62]

*95% BCa confidence intervals, based on 2,000 bootstrap samples, within brackets.*

Our key research question concerns how valuations of unselfishness and tolerance predict cooperative behavior, cooperative norms, and cooperative metanorms. Pairwise Pearson correlations are given in Table 4. The first row of the table shows that the cooperation index (the composite measure of behavior, norms, and metanorms) was strongly positively correlated with the valuation of tolerance but uncorrelated with the valuation of unselfishness. The other rows of the table confirms that this pattern was observed also for each component.

**TABLE 4:** Pearson correlations between valuations of personal qualities and measures related to cooperation.

**Dependent variable**	**N**	**Correlation with valuation of unselfishness**	**Correlation with valuation of tolerance**
Cooperation index	62	0.11 [−0.12, 0.34]	0.59 [0.46, 0.70]
Cooperative behavior in PD	40	0.18 [−0.12, 0.48]	0.53 [0.32, 0.72]
Cooperative norm in CPR	51	−0.05 [−0.28, 0.20]	0.37 [0.16, 0.57]
Metanorm against physical confrontation	51	0.08 [−0.16, 0.34]	0.59 [0.42, 0.76]
Metanorm in favor of gossip	51	0.03 [.−0.22, 0.28]	0.42 [0.19, 0.62]

*95% BCa confidence intervals, based on 2,000 bootstrap samples, within brackets.*

**FIGURE 1:**
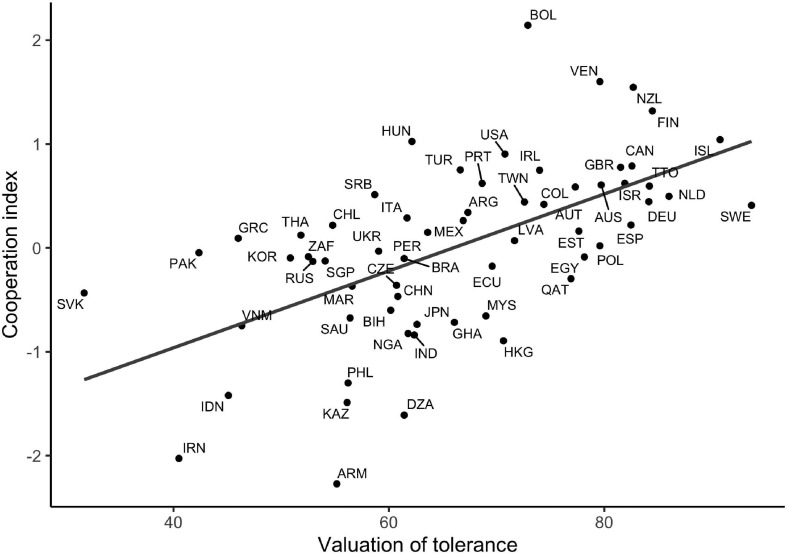
Scatter plot of the cooperation index against the valuation of tolerance in 62 societies, abbreviated according to the ISO three-letter standard. The plot shows that the cooperation index (a composite measure of cooperative behavior, norms, and metanorms) is higher in societies where tolerance is a more highly valued quality in persons; the regression line explains 34% of the variance.

Finally, we calculated correlations between the cooperation index and the other predictors of cooperation. As expected, all correlations were positive: *r* = 0.33 for absence of infectious diseases (*n* = 62), *r* = 0.48 for relational mobility (*n* = 31), *r* = 0.43 for median income (*n* = 60), *r* = 0.41 for indulgence (*n* = 59), and *r* = 0.28 for trust (*n* = 62). However, valuation of tolerance (*r* = 0.59) outperformed all predictors; see Figure 1 for a scatter plot illustrating this correlation. Similar conclusions about the predictive performance of valuation of tolerance hold if we instead look at each cooperative measure separately (see Supplementary Figure 1).

### Multi-Level Analysis Using Individual-Level Data From the International Study of Metanorms

To enable analyses at the individual level, we now switch the source of data on valuations of unselfishness and tolerance from WVS/EVS to the ISMN. A first observation is that the valuations of unselfishness and tolerance correlated very weakly at the individual level, *r* = 0.05, 95% CI [0.03, 0.06], *n* = 17,888. After aggregation of valuations to the society level, they were more strongly correlated, *r* = 0.33, 95% CI [0.08, 0.54], *n* = 57. Recall that this correlation was weaker in the WVS/EVS data reported above. As a first step we checked whether the findings of society level correlations with cooperative norms and metanorms replicated in the ISMN data. Consistent with the results reported in Table 4, the societies’ valuations of tolerance correlated with their cooperative norms, *r* = 0.44 (*n* = 57), with their metanorms against physical confrontation, *r* = 0.42 (*n* = 57), and with their metanorms in favor of gossip, *r* = 0.50 (*n* = 57), while the corresponding correlations with valuations of unselfishness were much weaker, *r* = 0.18, 0.23, and 0.15, respectively. Thus, the society level pattern of results was robust.

We now turn to an examination of whether the society level effects are accounted for by individual-level effects. To do this we estimate multiple mixed linear models with random intercepts at the society level. For each norm or metanorm we estimate two models, see Table 5. The first set of models (numbered 1, 3, and 5 in the table) include only the society level valuations of unselfishness and tolerance as predictors (of cooperative norms, metanorms in favor of gossip, and metanorms against physical confrontation, respectively). Using these models, we replicate the finding of positive effects of societal valuations of tolerance and null effects of societal valuations of unselfishness. The second set of models (numbered 2, 4, and 6) additionally include the individual-level valuations of unselfishness and tolerance, thereby partitioning the total society-level effect of each variable estimated in the first set of models into two parts: the portion that is a direct consequence of an individual-level effect and the remaining portion that is a genuine society-level phenomenon. As Table 5 shows, there were small positive individual-level effects of both valuations but nearly the entire society-level effect proved to be robust.

**TABLE 5:** Estimates from mixed linear models.

	**Cooperative norm**		**Metanorm in favor of gossip**		**Metanorm against physical confrontation**	
	**Model 1**	**Model 2**	**Model 3**	**Model 4**	**Model 5**	**Model 6**
Fixed effects						
Societal valuation of unselfishness	0.04	−0.07	−0.05	−0.05	0.24	0.16
	[−0.27, 0.35]	[−0.38, 0.23]	[−0.46, 0.35]	[−0.46, 0.35]	[−0.44, 0.91]	[−0.51, 0.84]
Societal valuation of tolerance	1.00	0.78	1.65	1.63	1.89	1.71
	[0.42, 1.57]	[0.20, 1.35]	[0.89, 2.41]	[0.88, 2.39]	[0.63, 3.16]	[0.44, 2.97]
Individual valuation of unselfishness		0.11		−0.00		0.07
		[0.08, 0.15]		[−0.03, 0.03]		[0.04, 0.11]
Individual valuation of tolerance		0.22		0.01		0.19
		[0.18, 0.26]		[−0.02, 0.05]		[0.14, 0.23]
Random part						
σ_*intercept*_	0.14	0.14	0.20	0.20	0.33	0.33
σ_*e*_	0.99	0.98	0.95	0.95	1.15	1.15
AIC	50517.2	50354.3	49075.5	49091.4	55985.6	55916.9

*Based on data from *n* = 17,888 participants from 57 countries who completed metanorm measures in the study of Eriksson et al. (2021).*

## Discussion

We linked data from recent large cross-cultural studies to examine two questions about how cooperation, cooperative norms and cooperative metanorms vary across societies. Our first question centered on the relation between cooperative behavior, norms, and metanorms. We hypothesized that cooperative behavior tends to be packaged with norms promoting cooperation and cooperative metanorms favoring gossip over physical confrontation. Consistent with this hypothesis, we found that societies with high levels of cooperation in a PD also had stricter norms against non-cooperation in a common pool resource scenario. Moreover, both behavior and norms were related in the predicted ways to metanorms about how to deal with non-cooperators. Thus, our results support theories emphasizing the role of norms for cooperative behavior as well as the importance of dealing with norm violators in ways that strengthen cooperative group norms and avoid within-group conflict.

Our second question concerned how cooperation depends on societies’ cultural emphasis on unselfishness and tolerance. We found cooperative behavior, cooperative norms, and cooperative metanorms were unrelated to societal valuations of unselfishness. For theories that view selfishness as the main obstacle to cooperation, this finding poses a conundrum. But we noted earlier that social heterogeneity and problems of trust may attenuate the link between a cultural emphasis on unselfishness and cooperation.

The main hypothesis of the present paper is that a tolerant culture may create less conflict and thereby be better able to realize high levels of trust and cooperation. Consistent with this hypothesis, we found societal valuation of tolerance strongly predicted societal levels of cooperation, as well as norms and metanorms supporting cooperation. Indeed, it was a better predictor than any other predictor suggested by prior research. Together, these findings suggest that cooperation is more closely tied to tolerance than to unselfishness at the societal level. The society level effect of tolerance values remained when we controlled for individual level values in a multi-level analysis. In support of our main hypothesis, these findings provide evidence that the main pathways linking tolerance values to cooperativeness reside at the societal level.

In addition to the society level effect, the multi-level analysis revealed small independent individual-level effects of valuing tolerance and unselfishness. Specifically, individuals who more strongly valued tolerance and unselfishness rated non-cooperative behavior as especially inappropriate. Assuming people who value tolerance and unselfishness are themselves tolerant and unselfish, these effects are unsurprising. The important novel finding is that these individual-level effects do not account for the society level effect. The interpretation is that cooperation is socially influenced by others’ valuations of tolerance.

Finally, a note about intercultural cooperation. The measure of cooperative behavior that we used was an aggregate of decisions in intercultural and intracultural settings (Romano et al., 2021). At the country level, these decisions were extremely strongly correlated across settings. In other words, the same country differences are found for intercultural cooperation as for intracultural cooperation. From the point of view of our main hypothesis, it makes sense that tolerance and respect for other people is important also in intercultural interactions.

## Conclusion

We have made several key contributions to the study of cooperation from a cross-cultural perspective. By demonstrating that cooperative behavior and cooperative norms tend to be coupled with specific cooperative metanorms (favoring gossip over physical confrontation in response to norm violators), we have validated and extended several long-standing theoretical arguments on cooperation. Moreover, by demonstrating that cooperative behavior, norms, and metanorms are better predicted by societies’ emphasis on tolerance than by their emphasis on unselfishness, we hope to contribute to a better understanding of the routes to human cooperation and the cultural determinants of more and less cooperative societies. Further theoretical and empirical research is required to tease out the pathways by which tolerance values are linked to the cooperativeness of a society.

## Data Availability Statement

The final dataset and analysis code are publicly available at https://github.com/irinavrt/tolerance-vs-unselfishness.

## Ethics Statement

Ethical review and approval was not required for this study of secondary data in accordance with the local legislation and institutional requirements.

## Author Contributions

KE and BS conceived the study and wrote the manuscript. KE and IV curated the data and performed the analyses. All authors contributed to the article and approved the submitted version.

## Conflict of Interest

The authors declare that the research was conducted in the absence of any commercial or financial relationships that could be construed as a potential conflict of interest.

## Publisher’s Note

All claims expressed in this article are solely those of the authors and do not necessarily represent those of their affiliated organizations, or those of the publisher, the editors and the reviewers. Any product that may be evaluated in this article, or claim that may be made by its manufacturer, is not guaranteed or endorsed by the publisher.

## Footnotes

1
http://www.worldvaluessurvey.org/

2
https://europeanvaluesstudy.eu/

3 Results are similar if we instead pool data from all years a country has participated.

4 At the national level, the cooperation measures for different treatments had excellent internal consistency, α = 0.99.

5
www.hofstede-insights.com/product/compare-countries/

6 Counting only the participants who completed all these measures.

## Supplementary Material

Supplementary Figure 1 Pearson correlations between cooperative measures and predictors of cooperation. Whether the cooperativeness of societies is measured by behavior, norms, or metanorms, the value of tolerance is consistently the strongest or second strongest among a wide range of predictors. Error bars signify 95% confidence intervals. Note that sample sizes vary across cooperative measures and predictors.
