# Supplementary figures and images for: The Power of Tolerance vs. Unselfishness as a Cultural Determinant of Cooperation
Source: Front Psychol. 2021 Sep 7;12:678237. doi: 10.3389/fpsyg.2021.678237 (PMC8452855; doi:10.3389/fpsyg.2021.678237)

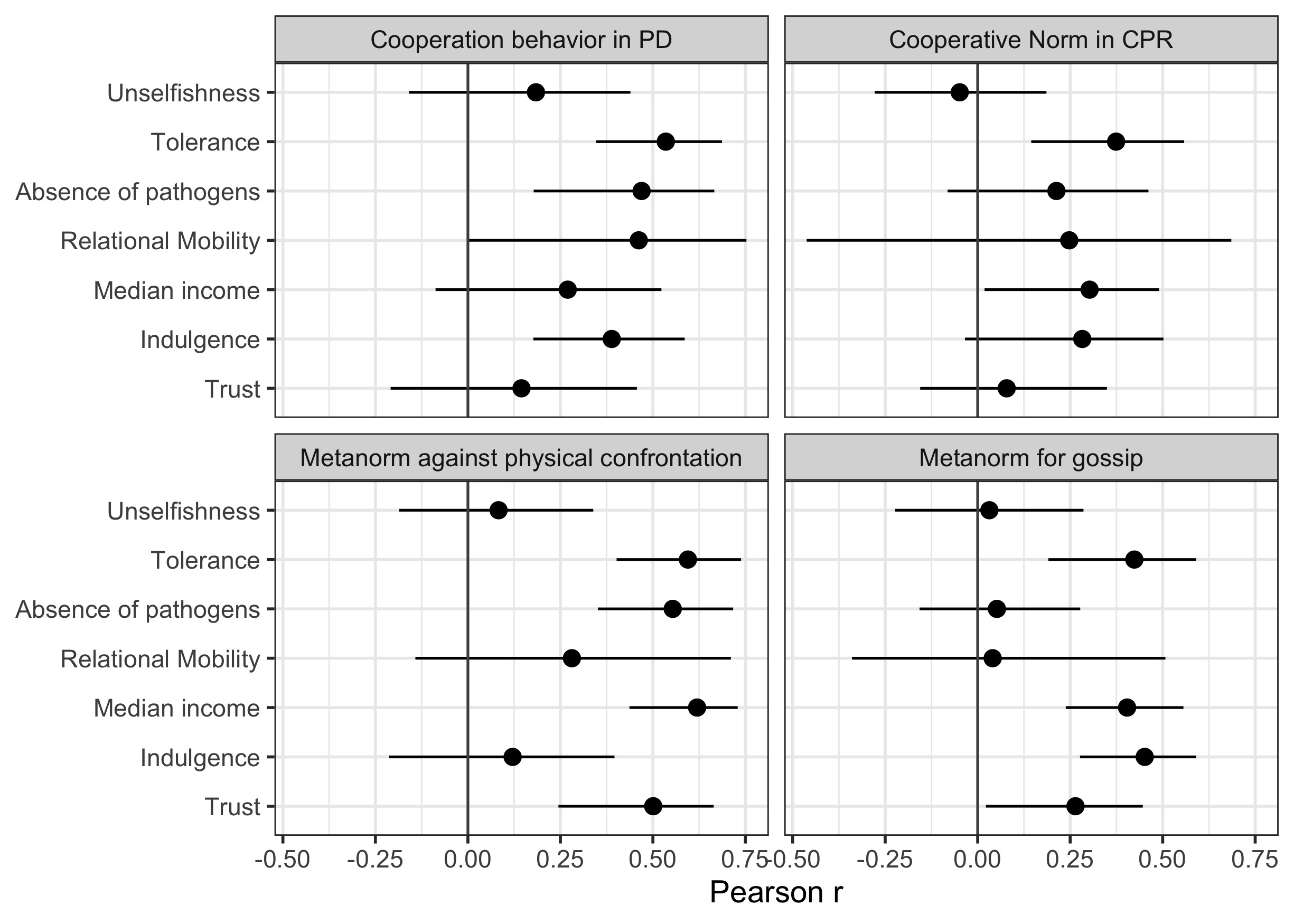

Supplement: Supplementary Figure 1 — Pearson correlations between cooperative measures and predictors of cooperation. Whether the cooperativeness of societies is measured by behavior, norms, or metanorms, the value of tolerance is consistently the strongest or second strongest among a wide range of predictors. Error bars signify 95% confidence intervals. Note that sample sizes vary across cooperative measures and predictors. [file Image_1.JPEG]
